# Could *Faecalibacterium prausnitzii* Amount in the Gut Microbiota Be Used to Monitor the COVID-19 Severity?

**DOI:** 10.5152/tjg.2022.22100

**Published:** 2022-10-01

**Authors:** Mehmet Demirci

**Affiliations:** 1 Department of Medical Microbiology, Kırklareli University Faculty of Medicine, Kırklareli, Turkey

Dear Editor,

The severe acute respiratory syndrome coronavirus 2 (SARS-CoV-2) pandemic is not over yet. It continues to cause concern worldwide with its emerging new variants. The immunopathogenesis mechanism and relationship with the disease severity in individuals are still not clearly understood. Coronavirus disease 2019 (COVID-19) severity is thought to depend on the host, virus, and environmental factors. Therefore, evidence that will enable us to detect new biomarkers to target COVID-19 severity may be more helpful in this battle. It is known that gut microbiota is associated with disease severity in SARS-CoV-2-positive patients.^1^ *Faecalibacterium prausnitzii *is an important bacterium with anti-inflammatory properties capable of producing short-chain fatty acids (SCFAs), such as butyrate, and is considered a new probiotic in the gut microbiota.^2^

When searched Pubmed and Pubmed Central on January 25, 2022, with the keywords “*Faecalibacterium*” and “COVID-19,” 480 articles were found, but only 4 of them investigated *F. prausnitzii* amounts in the gut microbiota between severe and non-severe COVID-19 patients. In these 4 articles, 215 non-severe and 88 severe (severe/critical) COVID-19 patients were analyzed for *F. prausnitzii* amounts in the gut microbiota.^1,3-5^ Regardless of age differences, *F. prausnitzii* was reported to have decreased in the gut microbiota of severe COVID-19 patients compared to non-severe COVID-19 patients.^1,3-5^ Figure 1 shows a comparison of *F. prausnitzii* in the gut microbiota reported in severe COVID-19 patients with those in non-severe COVID-19 patients. Reinold et al^3^ reported that proinflammatory cytokines such as interleukin-6 were high and the anti-inflammatory response was suppressed in severe COVID-19 patients compared to non-severe COVID-19 patients. This was correlated with a decrease in *F. prausnitzii* amounts in the gut microbiota. The “lung–gut axis” indicates bidirectional communication, and it appears that SCFAs-producing bacteria in the gut microbiota play an important role in the inflammatory response in the lungs. We obtained data supporting the lung–gut axis effect of *F. prausnitzii* in the gut microbiota of children with asthma^2^

To conclude, *F. prausnitzii* might be declining in the gut microbiota of severe and critical COVID-19 patients. The monitoring of the *F. prausnitzii* amount in the gut microbiota using the quantitative polymerase chain reaction method can be evaluated to follow-up disease severity. In addition, the follow-up of this bacterium in donor stools and the standardized use of this bacterium in fecal microbiota transplantation could be considered in the treatment of severe and critical COVID-19 patients. On the other hand, due to both limited studies and difficulties in comparing results, definite conclusions cannot be drawn. The decline of *F. prausnitzii* abundance in the gut microbiota may be innocent. Prospective follow-up is required. In addition, some clinical trials investigating the safety of *F. prausnitzii* in case of therapeutic use are ongoing; it should be approached carefully until its safety is confirmed.

## Figures and Tables

**Figure 1.**
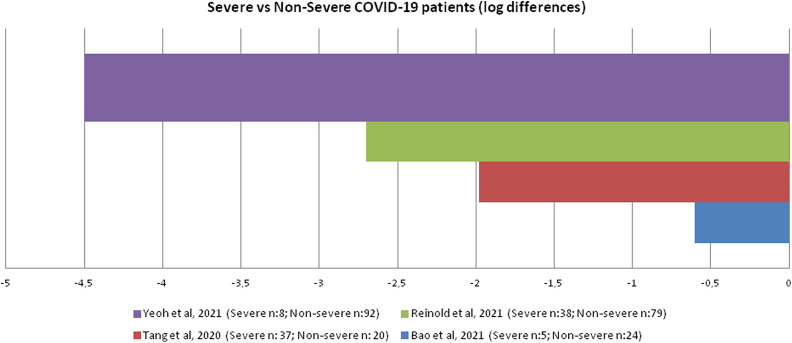
Comparison of *Faecalibacterium prausnitzii *in the gut microbiota reported in severe COVID-19 patients with those in non-severe COVID-19 patients. COVID-19, coronavirus disease 2019.
